# Evaluating the utility of a patient and public involvement and engagement (PPIE) end-of-trial event to re-engage with cell-based therapy participants

**DOI:** 10.1080/17460751.2025.2601546

**Published:** 2025-12-18

**Authors:** Deepak Menon, Jan-Herman Kuiper, Andrea Bailey, Michael Murphy, Yumna Ladha, Peter Gallacher, Paul Jermin, Andrew Roberts, Geraint Thomas, Charlotte H. Hulme, Karina T. Wright

**Affiliations:** a Centre for Regenerative Medicine, School of Medicine, Keele University, Keele, UK; b Oswestry Keele Orthopaedic Research Group (OsKOR), Robert Jones and Agnes Hunt Orthopaedic Hospital NHS Foundation Trust, Oswestry, UK; c Centre for Regenerative Medicine, School of Life Sciences, Keele University, Keele, UK

**Keywords:** Cell-based therapy, patient perspectives, patient and public involvement and engagement (PPIE), autologous chondrocyte implantation, stem-cell implanation, cartilage injury

## Abstract

**Aims:**

Although research in cell-based therapy is expanding, little is known about patient experience in cell therapy trials to-date. This study explores the experience and attitudes of participants involved in the ASCOT randomized controlled trial, a long-term orthopedic study comparing different cell therapies for the treatment of knee articular cartilage defects.

**Methods:**

The clinical and rehabilitation experience of participants, and their attitudes relating to i) patient and public involvement (PPIE) in research and ii) future research on cellular therapy and clinical trials were explored through questionnaires. To gather this information, the utility of an end-of-trial event to reengage participants was evaluated.

**Results:**

Despite the anticipated high burden, overall trial experience was reported as highly positive. Communication and ongoing engagement between patients and both the clinical and research teams may have contributed to this. Key challenges identified for future research include the need for early and transparent communication regarding trial results, and greater support to facilitate active PPIE involvement. The end-of-trial engagement event demonstrated feasibility and value as a model to gather this important PPIE data for future studies.

**Conclusions:**

This study highlights the importance of integrating participant perspectives to optimize trial design and delivery in future cell therapy research.

## Plain Language Summary

Research and delivery of cell-based therapies is growing quickly. Cell-based therapies use living cells, often to repair or replace damaged tissues. For these treatments to become widely available, they have to be tested in clinical trials. However, we still know little about what it is like for patients to take part in clinical trials using cell-based therapies. This study explored the experiences and attitudes of people involved in the ASCOT trial, a long-term study comparing different cell therapies for repairing damaged cartilage.

Participants were asked about their experience of taking part in the study, their rehabilitation experiences and to share their views on future cell therapy trials. Information about how to best design and deliver future research which embeds patient and public involvement (known as PPIE) was also gathered. To collect this feedback, the research team tested whether an end-of-trial event would be a good way to reconnect with participants.

Although the clinical trial required significant time and effort, including long follow-up time and completion of many follow-up questionnaires, participants reported very positive experiences overall. Participants also highlighted opportunities for improvement in future trials, such as providing earlier and more transparent updates on trial results and offering better support to help patients take an active role in PPIE.

The study showed that an end-of-trial event is a useful and practical way to gather this kind of feedback. Overall, the findings emphasize how important it is to include participant perspectives when designing and running future cell-therapy research.

## 1. Introduction

Symptomatic localized articular cartilage defects of the knee may be treated with various cell-based therapies. The two main cell types considered for this indication are chondrocytes and bone marrow derived, however, there is no consensus as to whether bone marrow-derived stem cells (BMSCs) or chondrocytes are superior in the treatment of this pathology. The ASCOT (Autologous Stem Cells, Chondrocytes, Or the Two?) trial is a single-center, single-blinded randomized controlled trial that aims to compare clinical outcomes of autologous stem cell implantation (ASI, using BMSCs), autologous chondrocyte implantation (ACI) and a combination of both (ASCI) in the treatment of chondral or osteochondral knee defects, as described previously [1]. The final trial participant was recruited in 2023. Given a follow-up period of 15 months, initial trial results are expected to be reported in 2025/2026.

This clinical trial aimed to identify the best cultured cell therapy option, such that clinical outcomes and quality of life of individuals with symptomatic articular defects of the knee were the most improved. In parallel with the trial, a significant body of scientific work aimed to determine the characteristics of the patients and their cells that were associated with beneficial clinical and cartilage repair outcomes, requiring donation of multiple patient samples. Being a trial participant in this study required significant patient input. Pre-operatively, all individuals required a diagnostic MRI, baseline CT and MRI scan, and completed a series of patient-reported outcome measure (PROM) questionnaires to determine quality of life, knee function, activity levels and mood. Intervention included two major staged surgical procedures. In addition, those in the ACI group required a 5 mm incision in the region where bone marrow biopsy would be taken (in the absence of bone marrow aspiration) to maintain blinding. Post-operatively, all patients underwent rehabilitation with physiotherapy engagement in addition to requiring a further CT, MRI and arthroscopy between 12 and 15 months following initial surgery to determine the quality of the repair tissue. During the post-operative arthroscopy, a tissue biopsy of the tissue harvest site and repair site was obtained. There has been a prolonged recruitment period of 9 years from 2014 to 2023. During this period, trial participants have been asked to complete extensive PROMs annually. Those who were recruited early in the study time-period have therefore had limited contact with the study site or update regarding trial progress, other than completion of PROMs.

Understanding patient experience in complex trials such as ASCOT is vitally important for learning and improvements in future trial and scientific study design. Validated surveys have been developed to understand and drive improvements in participant experience [2,3]. However, there is limited literature exploring patient experience in trials involving cell-based therapy which are associated with unique challenges. Cell-based therapies utilize the transfer of living cells to treat disease [4]. They are approved and regulated by the Medicines and Healthcare products Regulatory Agency (MHRA) in the UK, European Medicine Agency in Europe [5] and the Center for Biologics Evaluation and Research in the US [6]. Regulatory, delivery, social, health and economic challenges exist for cell therapies, which require understanding and input from patients to ensure their benefits can be successfully delivered. In addition to exploring trial participant experience, evaluating patient and public attitudes toward future research in the field of cellular therapy is important to strengthen the quality and translational relevance of the output. Notably, Aiyegbusi et al. emphasized the importance of improving patient and public engagement and understanding of cell therapies to secure societal and government support for cell therapy development [7]. This is particularly important as cellular therapies have unique ethical and logistical challenges as well as risks compared with other pharmacological agents; many misconceptions and much misinformation relating to these are being identified [7]. This indicates the importance of engaging with patients at each stage of the research and trial process, to ensure patient facing information is made understandable and without adding to any misconceptions. Moreover, they highlighted that at the time no studies had examined the experiences of individuals who had received cell or gene therapies [7].

There is now a drive to engage patients and public members in all aspects of clinical trials including protocol development, defining outcomes and running studies [8]. PPIE involvement in trials has been shown to have a positive impact on enrollment rate [9] as well as the quality of outcomes generated [10]. There is therefore a need to maintain this patient-centered approach when developing clinical trials involving cell-based therapy.

We hypothesize that an event held at the end of a clinical trial recruitment process is feasible, useful to reengage with cell-based therapy clinical trial participants and provides a platform to gather information on trial experiences. Our study therefore aims to present the process and assess the utility of an end-of-trial participant event. Specific objectives are to: i) explore the clinical experience of participants involved in the ASCOT clinical trial, ii) summarize attitudes relating to future scientific research with a focus on cartilage repair, cellular therapy and clinical trials, iii) appraise participants’ rehabilitation experiences, and iv) highlight attitudes relating to the involvement of patients and the general public in future clinical trial design and delivery.

## 2. Materials and methods

Local institutional approval (ID 2324_020) from the ASCOT clinical trial study sponsor (The Robert Jones and Agnes Hunt Orthopaedic Hospital NHS Foundation Trust) was obtained to run this project as a service evaluation, which involved recontacting all trial participants and to run an end-of-trial event to assess perspectives on trial experience and scientific research through a questionnaire-based methodology. Formal research ethics committee (REC) approval was not required. This study was performed in line with the principles of the Declaration of Helsinki.

### 2.1. Trial participant engagement event

All 114 ASCOT trial participants were identified. Participants who had not withdrawn their consent for contact were contacted (*n* = 98) via telephone by two members of the trial team. Over the phone, the opportunity to attend an ‘ASCOT Trial Celebration’ event was presented. Other members of the public (relatives, friends and carers) were encouraged to attend. If participants were interested, an electronic mail (e-mail) address was provided, and an invitation was sent to this one month prior to the event. In cases where trial participants were unable to be contacted, a flyer with information about the event was sent to their recorded address. At least three attempts were made to contact trial participants before they were deemed uncontactable. Participants were offered a voucher to support the costs of travel associated with attending the event.

The event was held at the trial clinical center and split into three sessions focusing on the following themes: i) Trial Development and Delivery ii) Trial Outcomes Measures and iii) Scientific Investigations. Sessions were delivered by a multidisciplinary team including scientists, managers, surgeons, radiologists and physiotherapists and included an opportunity for an open-forum discussion between trial participants and members of the trial team (Table 1). Participants were given a tour of the trial center, including the cell manufacturing facilities, with several opportunities for open interaction between the participants and the trial team via regular refreshment breaks.

**Table 1.** Structure and delivery of the ‘ASCOT trial celebration’ event.

Session 1: Trial Development and Delivery	Background to the ASCOT Trial (ACI and MSCs) Trial Design Session Discussion and Clinical Trial Questionnaire
Session 2: Trial Outcome Measures	Patient Reported Outcome Measures: The Importance of Long-Term Outcomes MRI and CT Imaging Cartilage Biopsies Rehabilitation Session Discussion and Rehabilitation Questionnaire
Session 3: Scientific Investigations	Scientific Objectives Careers Launched/Supported Session Discussion and Scientific Questionnaire

Feedback was sought using questionnaire-based methodology. The satisfaction of each session, tour of the facilities, and organization of the event were rated on a ten-point scale (Supplementary File, S1).

### 2.2. Patient perspectives on trial experience and scientific research

The authors were also involved in the development of three questionnaires. A ‘Trial Participant Clinical Experience Questionnaire’ was initially developed (Supplementary File, S2), consisting of two sections. The first section was based on the validated Research Participant Perception Survey (RPPS) [11] with questions aiming to understand the reasons why ASCOT trial participants joined the study, individual experiences pertaining to the consent process and interactions with the research staff, and a subjective evaluation of study demand and the overall study experience. The second section contained questions aiming to evaluate trial participant attitudes relating to the involvement of patients and the general public in the design of clinical trials (in general) and dissemination of trial results. A ‘Trial Participant Perspectives for Translational Scientific Research Questionnaire’ contained seven statements which aimed to gauge individuals’ perspectives on scientific research relating to cellular therapy, cartilage repair and early osteoarthritis in addition to experimental treatments or clinical trials (Supplementary File, S3). Finally, the ‘Research Participant Rehabilitation Experience Questionnaire’ contained questions aiming to understand the rehabilitation-specific experiences of trial participants who were engaged in the ASCOT clinical trial, split into pre-operative and post-operative experience sections (Supplementary File, S4). The questionnaires employed a range of evaluation metrics to capture both quantitative and qualitative data on participant perspectives and experiences. These included scales for quantifying attitudes and satisfaction levels (including Likert-type scale and Ten-point scale), categorical and dichotomous (Yes/No) choices and free-text responses. For example, the evaluation of participant attitudes relating to PPIE in trial design and trial result dissemination included four questions requiring responses on a Likert-scale, one dichotomous Yes/No question, and one question requiring a free-text response (Supplementary File, S2).

Questionnaires were distributed to all ‘ASCOT Trial Celebration’ event attendees as part of the session discussions (Table 1). Following initial telephone contact where consent was obtained, questionnaires were also distributed to all ASCOT trial participants who did not attend the event by either e-mail or post. Informed consent information was provided and obtained from participants completing the questionnaires. Anonymity was assured for the survey data used in analysis, though personal identifiers were used to cross-reference to demographic and clinical data.

### 2.3. Statistical analysis

All analyses were performed using R version 4.5.2. Categorical variables were summarized as frequencies and percentages, and Pearson’s Chi-squared or Fisher’s exact test was used for hypothesis testing depending on expected cell counts. Continuous variables were summarized as mean with standard deviation (SD) or medians with interquartile range (IQR) depending on data distribution, which was assessed using QQ plots and histograms. Hypothesis testing was performed using the independent samples t-test, Mann-Whitney U test or Kruskal-Wallis test depending on data distribution. A p-value < 0.05 was defined as statistically significant. Graphical presentation (bar charts and frequency plots) was used to illustrate responses to key questionnaire items.

## 3. Results

### 3.1. Trial participant engagement event

Thirty-one (27.2%) trial participants confirmed participation prior to the engagement event with eight additional guests. Twenty-one trial participants with seven additional guests attended the event. Details of all trial participants who attended the event were cross-referenced to the ASCOT clinical trial data. Baseline demographics are presented in Table 2. No statistically significant differences were observed between trial participant event attendees and non-attendees with respect to demographic factors or time from event to date of surgery (Table 2).

**Table 2.** Baseline demographics and clinical characteristics of ASCOT trial participants stratified by engagement event attendance and questionnaire completion.

		Engagement event attendees (*n* = 21)	Engagement event non-attendees (*n* = 89)	*p*-value	Questionnaire respondents (*n* = 26)	Questionnaire non-respondents (*n* = 84)	*p*-value
Gender	Female	6 (28.6%)	30 (33.7%)	0.85	9 (34.5%)	27 (32.1%)	1.00
	Male	15 (71.4%)	59 (66.3%)		17 (65.4%)	57 (67.9%)	
Mean Age (SD) at Surgery		40.7 (9.5)	36.8 (8.4)	0.10	39.5 (9.3)	37.0 (8.5)	0.23
Smoking Status	Current	2 (9.5%)	10 (11.2%)	0.91	3 (11.5%)	9 (10.7%)	0.70
	None	16 (76.2%)	59 (66.3%)		20 (76.9%)	55 (65.5%)	
	Ex	3 (14.3%)	18 (20.2%)		3 (11.5%)	18 (21.4%)	
	Unknown	0 (0.0%)	2 (2.2%)		0 (0.0%)	2 (2.4%)	
Median BMI (kg/m^2^)		30.0 (IQR 27.0 – 33.0)	28.0 (IQR 25.0 – 33.0)	0.83	29.0 (IQR 27.0 – 32.0)	29.0 (IQR 25.0 – 33.0)	0.94
Median time from index date to surgery (days)*		2620 (IQR 1870 – 2947)	2299 (IQR 1745 – 3140)	0.93	2414 (IQR 1874 – 2904)	2338 (1742 – 3149)	0.84

*Index Date = 1st December 2023; Surgery = Date of 2nd Procedural Stage.

Eleven individuals completed event-specific feedback. Median satisfaction score was 10.0 [IQR 9.5 – 10.0], 10.0 [IQR 9.0 – 10.0] and 10.0 [IQR 9.0 – 10.0] for each of the ‘Trial Development and Delivery,’ ‘Trial Outcome Measures,’ and ‘Scientific Investigations’ sessions respectively. In addition, median satisfaction scores for the trial center tour, event organization and response to the question ‘Overall, did you find the celebration to be an enjoyable and engaging experience?’ were 10.0 [IQR 9.0 – 10.0], 10.0 [IQR 10.0 – 10.0] and 10.0 [IQR 10.0 – 10.0] respectively. All free text comments provided relating to the engagement event sessions and event overall are presented in Table 3.

**Table 3.** Summary of free-text comments from ‘ASCOT trial celebration’ event attendees.

Session 1	Session 2	Session 3	Other comments
*“ … really enjoyed all of the content and explanation of the trial. Would have loved to have seen some videos of the surgery.”*	*“[Would] be good to see the scan results for different treatments.”*	*“Well presented and informative.”*	*“ … it was very informative and interesting … good to meet the other people involved in my treatment.”*
*“Informative and a good insight.”*	*“Some of the radiologist information was quite technical for [the] lay person.”*	*“Nice to know the benefits and follow up stories, not just [that the trial was] helping patients.”*	*“[It would] be nice to see what achievements people have done and compare fitness schedules … [to] see how it helps the knee.”*
*“Well presented.”*	*“Helped me understand more [about] the science … .”*		*“Loved the whole day [and] would love there to be another when the results are published.”*
*“Good overview of [the] trial … ”*	*“Very good.”*		*“Great to meet the people behind the trial and have a deeper understanding of the methodology and science. Also great to hear from other patients.”*
*“Really useful and gave a good insight into the ASCOT Trial.”*			*“It was nice to hear it was a learning curve for the physiotherapist as well; hard work especially as I didn’t always have contact … as [I] lived 3 hours away.”*

### 3.2. Patient perspectives on trial experience and scientific research – questionnaire results

#### 3.2.1. Participants

Twenty-six (22.8%) trial participants completed the ASCOT Trial Participant Clinical Experience Questionnaire, Rehabilitation Experience Questionnaire and Trial Participant Perspectives for Translational Scientific Research Questionnaire. Details of all individuals who completed the questionnaires were cross-referenced to the ASCOT clinical trial data. Baseline demographics of the respondents are presented in Table 2. No statistically significant differences were identified between trial participants who completed and did not complete the questionnaires. In addition, no significant difference was identified in time from event to date of surgery (Table 2). Of those who completed the questionnaires, twenty (76.9%) attended the engagement event, and all respondents stated they were of ‘White’ ethnicity.

Median pre-operative Lysholm Knee Score of the respondents’ affected knee was 13.0 (IQR 9.0 – 15.0). Median post-operative Lysholm Knee Score of the respondents’ affected knee at 15 months follow-up was 21.0 (IQR 16.0 – 21.0). For comparison, median pre-operative and 15-month post-operative Lysholm Knee Scores of all ASCOT trial participants were 11.0 (IQR 9.0 – 15.0) and 18.0 (IQR 14.0 – 21.0) respectively. These results are provided only for contextual background regarding the functional status of questionnaire respondents and not for the analysis of clinical efficacy.

#### 3.2.2. Clinical experience of ASCOT trial participants

**Figure 1.**
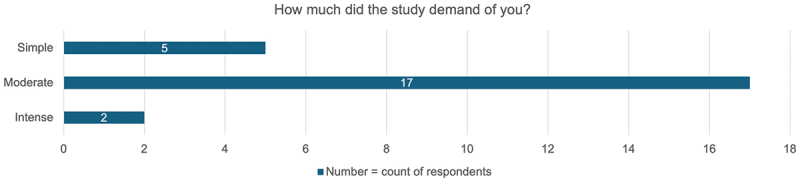
Self-reported demand of the ASCOT trial on participants.

**Figure 2.**
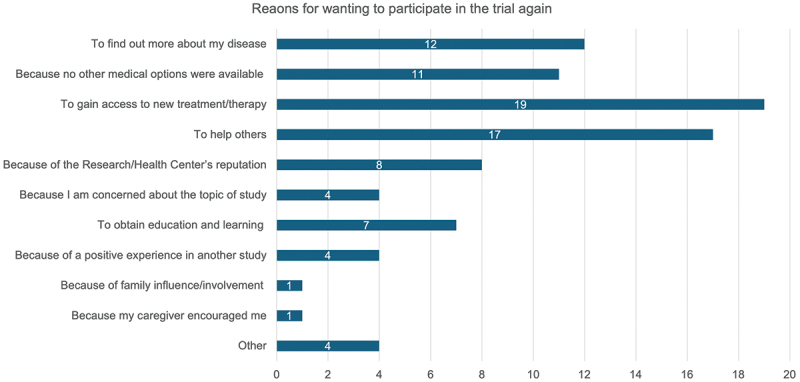
Participants’ top three reasons for willingness to re-participate in the ASCOT trial.

**Figure 3.**
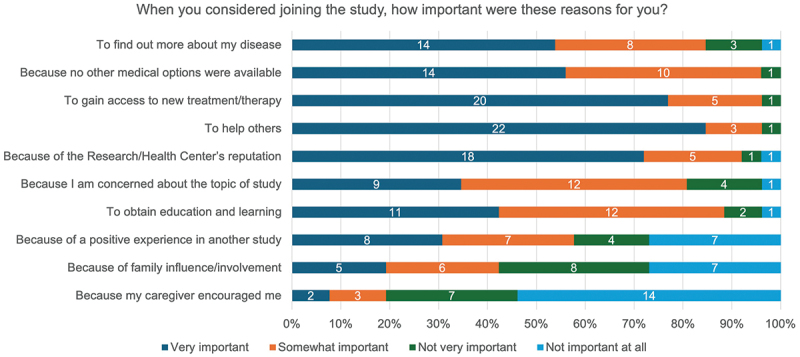
Importance of factors considered by participants when joining the ASCOT clinical trial.

Seventy-one percent (17/24) of respondents reported the study was ‘Moderate’ with respect to its demand on trial participants (Figure 1). Despite this, the overall median trial experience was 9.0 (IQR 8.3 – 10.0) rated on a ten-point scale and all respondents stated they would participate again if given the opportunity (84.6% [22/26] definitively re-participating and 25.4% [4/26] probably re-participating). No significant differences in satisfaction scores were observed when stratified by demographics (Table 4) or when comparing engagement event attendees and non-attendees (attendees [*n* = 20]: median score 9.0 [IQR 8.8 – 10.0], non-attendees [*n* = 6]: median score 9.0 [IQR 8.3 – 9.8]) (Table 4). All respondents stated they would recommend joining a research study to their family and friends (73.1% [19/26] definitively recommending and 26.9% [7/26] probably recommending). The top three reasons for wanting to participate again in the trial were to be able to access a new treatment, to help others, and to find out more about the disease process (Figure 2). This aligns to the reasons trial participants reported for joining the clinical trial (Figure 3).

**Table 4.** Satisfaction scores reported by questionnaire respondents stratified by demographics.

		Median Satisfaction Score	p-value
Gender	Male	9.0 (IQR 8.8 - 10.0)	0.38
	Female	10.0 (IQR 8.8 - 10.0)	
Age	20 to 29	8.5 (IQR 7.8 - 9.3)	0.46
	30 to 39	10.0 (IQR 9.0 - 10.0)	
	40 to 49	9.0 (IQR 8.5 - 10.0)	
	≥50	9.0 (IQR 8.5 - 9.3)	
Smoking Status	Current	8.5 (IQR 8.3 - 8.8)	0.53
	None	9.0 (IQR 9.0 - 10.0)	
	Ex	10.0 (IQR 8.5 - 10.0)	
BMI	Underweight (<18.5)	N/A	0.13
	Healthy (18.5 to 24.9)	7.5 (IQR 7.0 - 8.5)	
	Overweight (25 to 29.9)	9.0 (IQR 9.0 - 9.5)	
	Obese (≥30)	10.0 (IQR 9.0 - 10.0)	

**Figure 4.**
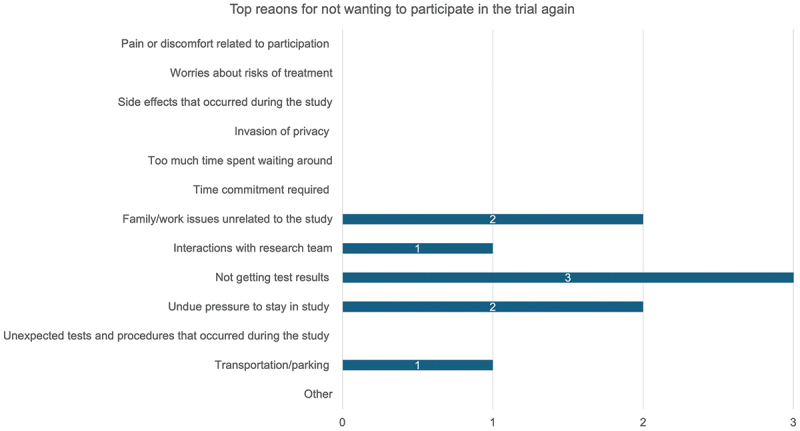
Participants’ top three reasons for not wanting to re-participate in the ASCOT trial.

**Figure 5.**
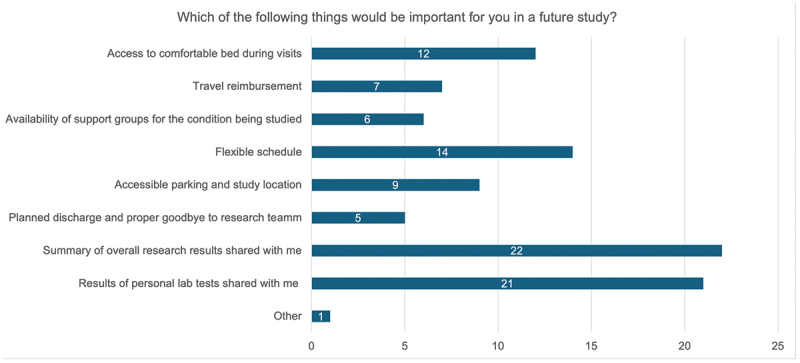
Factors rated as important for ASCOT trial participants when considering future trial participation.

The most common reason for not wanting to re-participate in the trial was due to not being provided with test results (Figure 4). This closely aligns to the factors considered most important in a future study, with the top three reasons being ‘summary of overall research results shared with me,’ ‘results of personal lab tests shared with me’ and ‘flexible schedule’ (Figure 5).

**Figure 6.**
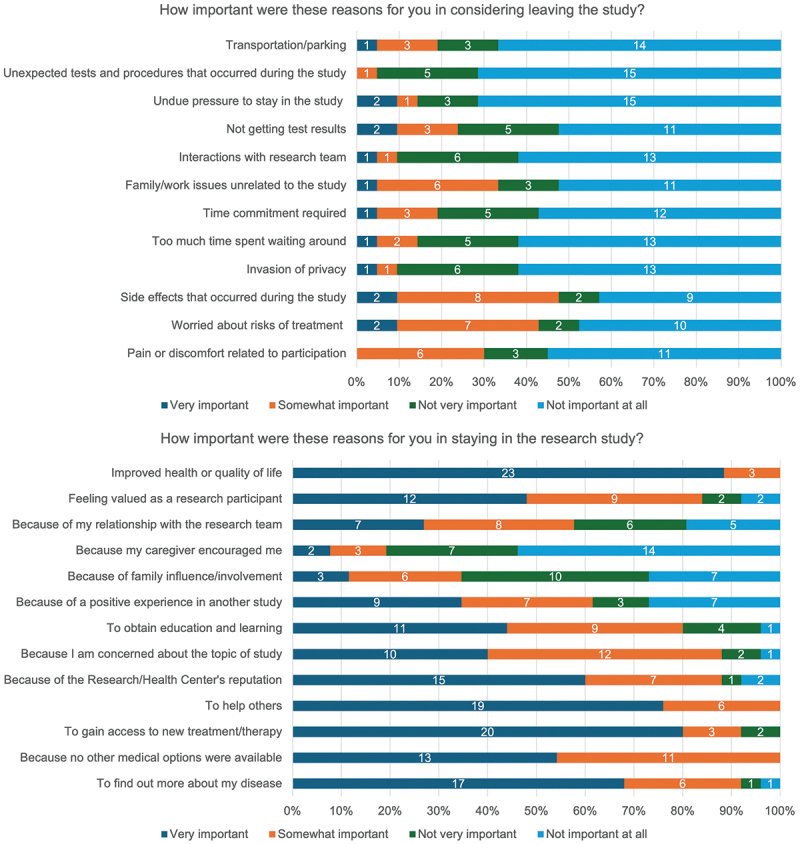
Participant perceptions on the importance of factors influencing the decision to leave and stay in the ASCOT trial.

The reasons for considering leaving and staying in the study are presented in Figure 6. The most important reasons for staying closely correlate to those important reasons indicated when individuals considered joining the study (Figure 4), with the notable inclusion of ‘improved health or quality of life’ as a key reason for continuing as a trial participant. Pain, side-effects and risks of treatment are noted to be key factors when trial participants considered leaving the study.

Individual anonymized responses to the clinical experience of ASCOT Trial Participants as part of the Trial Participant Clinical Experience Questionnaire are presented as a Supplementary File, S5.

#### 3.2.3. Rehabilitation experiences of ASCOT trial participants

**Figure 7.**
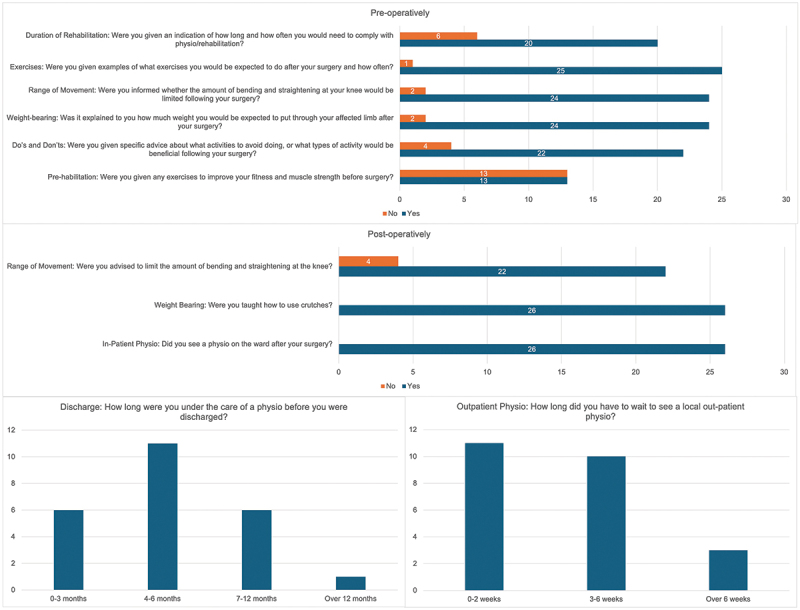
Pre-and post-operative rehabilitation experience of ASCOT trial participants.

The Trial Participant Rehabilitation Experience Questionnaire aimed to investigate both the pre- and post-operative rehabilitation experiences of trial participants. Pre-operatively, only 50.0% (13/26) were provided with exercises for prehabilitation prior to surgery and 76.9% (20/26) were given advice regarding expected duration of physiotherapy or rehabilitation. High rates of advice pertaining to post-operative exercises, range of movement, weight-bearing and ‘dos and don’ts’ were noted (Figure 7).

Post-operatively, all trial participants engaged with inpatient physiotherapy and were provided with education regarding crutch use. 18.2% (4/26) were not provided with advice to limit range of motion at the knee. 12.5% (3/24) waited over six weeks to see local physiotherapy as an outpatient, with the majority of individuals (45.8% [11/24]) discharged from physiotherapy care after four to six months of therapy (Figure 7).

Individual anonymized responses to the Trial Participant Rehabilitation Experience Questionnaire are presented as a Supplementary File, S5.

#### 3.2.4. Attitudes on future scientific research

**Figure 8.**
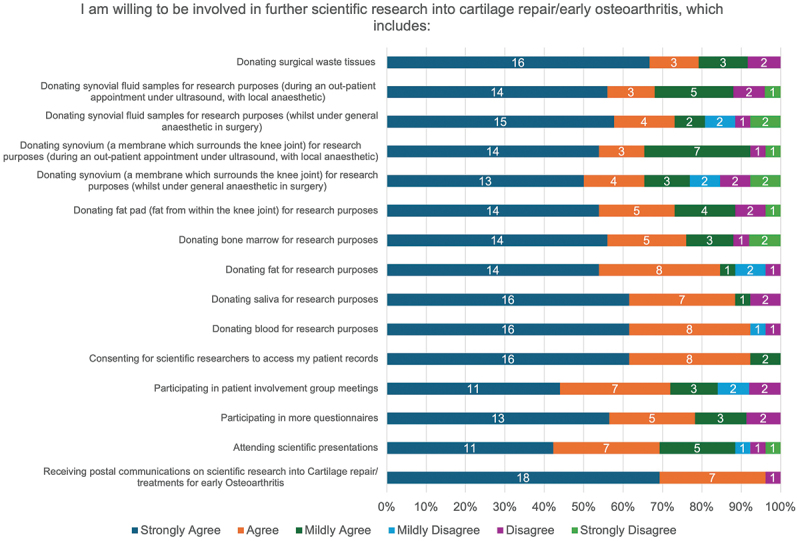
ASCOT trial participants’ willingness to be involved in future scientific research activities relating to cartilage repair or early osteoarthritis.

The majority (>50%) of questionnaire respondents indicated they would ‘Strongly agree’ to being involved in all activities when considering engagement in further scientific research into cartilage repair or early osteoarthritis, with the exception of attending scientific presentations (42.3% [11/26]) and participating in patient involvement group meetings (42.3% [11/26]) (Figure 8). The highest disagreement rate was associated with invasive procedures (donating synovium [23.1%, 6/26] or synovial fluid samples [19.2%, 5/26] under general anaesthetic) and active participant involvement in participating in patient group meetings (16.0% [4/25]). All individuals would agree with consenting for researchers to access patient records (Figure 8).

**Figure 9.**
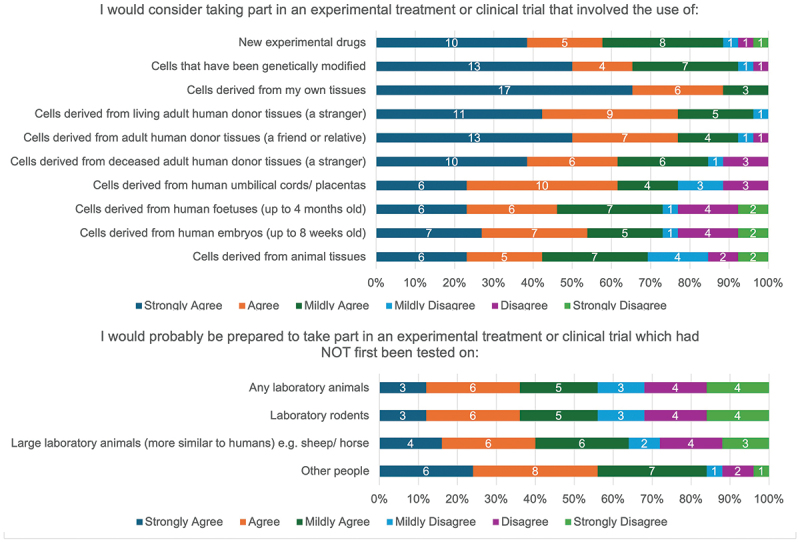
ASCOT trial participants’ willingness to engage in future experimental treatments or trials.

Most (>50%) questionnaire respondents indicated they would either ‘Agree’ or ‘Strongly agree’ to being involved in all types of experimental treatment, with the exception of those involving the use of animal tissues (42% [11/26]) and human fetuses (46.2% [12/26]) (Figure 9). High rates of disagreement were also noted when considering involvement in an experimental treatment or trial that had not first been tested on animals (Figure 9).

**Figure 10.**
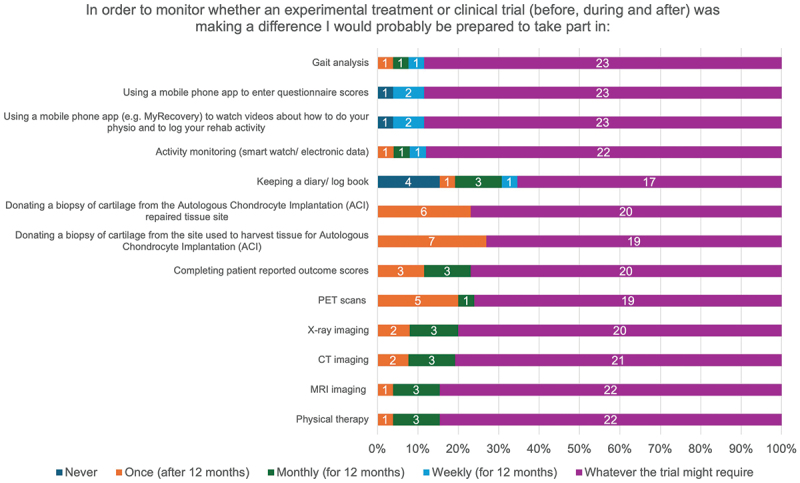
ASCOT trial participants’ willingness to engage in monitoring investigations as part of a future clinical trial.

With respect to monitoring investigations during an experimental treatment or clinical trial, most individuals would undertake whatever the trial required, though keeping a diary or logbook was associated with the highest number of ‘Never’ responses (4/26) (Figure 10).

Individual anonymized responses to the Trial Participant Perspectives for Translational Scientific Research Questionnaire are presented as a Supplementary File, S5.

#### 3.2.5. Attitudes on involvement of patients and the general public in future trial design and delivery

**Figure 11.**
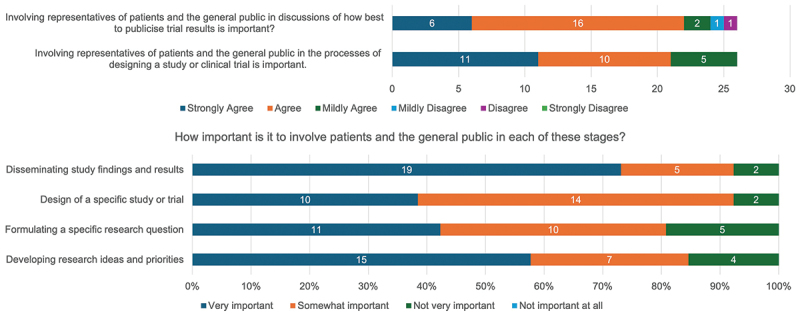
ASCOT trial participant attitudes on the importance of public and patient involvement and engagement in clinical trials.

Eighty-one percent (21/26) and 85% (22/26) of trial participants reported they would either ‘Strongly agree’ or ‘Agree’ that involving representatives of the general public or patients in processes relating to study design and dissemination of results in clinical trials is important (Figure 11). Involvement of patients and the public in the dissemination of study results and development of research ideas appears to be the priority area relative to involvement in study design and research question development (Figure 11).

**Figure 12.**
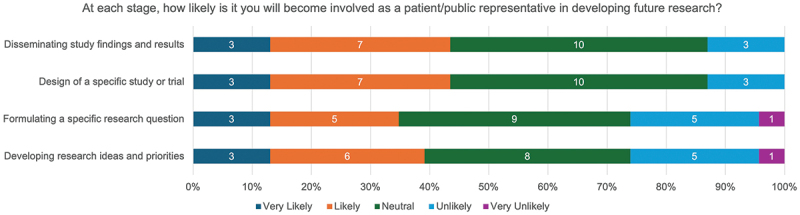
ASCOT trial participant likelihood of becoming involved as a patient or public representative in future research development.

However, 28.0% (7/25) of respondents stated they themselves would not like to be involved as a patient or public representative in developing future research, with the majority of questionnaire respondents (>50%) reporting they were ‘Neutral,’ ‘Unlikely’ or ‘Very unlikely’ to be involved as a representative in developing a research idea, formulating a research question, designing a study or trial, or disseminating research findings (Figure 12).

Individual anonymized responses to the attitudes of participants in future trial design and delivery as part of the Trial Participant Clinical Experience Questionnaire are presented as a Supplementary File, S5.

## 4. Discussion

This experiential study assesses the utility of an end-of-trial engagement event, as well as the experiences of participants involved in the ASCOT clinical trial, addressing the lack of published data on patient experiences in relation to cell-based therapy trials. It also investigates the attitudes of these individuals relating to i) future scientific research in the field of cartilage repair, and ii) PPIE in clinical trial design and delivery.

The importance of communicating and sharing study findings to trial participants following completion of a clinical trial is recognized, with guidance provided by the NHS Health Research Authority on how to facilitate this [12]. Methods include sharing newsletters or information sheets, social media posts, and verbal communication at study visits [12]. Patient engagement events are another option. These have been shown to improve adherence to intervention during clinical trials [13] and improve patient retention during long-term studies [14]. It is likely these events are also useful following trial completion to communicate progress/results, improve overall participant experience and promote re-participation in future studies. Our experience supports the feasibility and value of undertaking an end-of-trial participant engagement event. Median satisfaction scores were high across all delivered sessions and activities, and when rating overall organization, enjoyment and engagement. In addition, free-text comments were provided suggesting individuals appreciated the opportunity to meet other patients to discuss shared experiences, and that the event satisfied the objectives of updating trial participants of current study progress and generating excitement around the upcoming study results.

It is important to recognize that these findings should be taken in the context of the ASCOT study, which is a long-running trial with a prolonged recruitment period. Many participants, particularly those recruited early in the trial course, had limited contact with the study team and may therefore have appreciated the opportunity to reengage with the study team. Strategies such as increased study team contact, providing a forum for feedback and questions, and involvement of family have been demonstrated to improve participant experience in long-term studies [14]. This may contribute to the high levels of satisfaction reported in our study, which may not be reflected in similar events conducted in trials with a shorter recruitment and follow-up period. Interestingly, however, no significant difference was identified in the time from surgery between attendees and non-attendees. This may suggest participant interest in PPIE events remains consistent across the entire trial timeline and may not be affected by the length of follow-up. Furthermore, only a proportion of the total trial participants attended the engagement event which suggests further strategies are required to increase uptake and may indicate selection bias to those individuals who benefited the most from the trial being willing to reengage. Broadening accessibility by reducing the burden of travel and through digital technology has been advocated to improve engagement, retention and diversity in clinical trials [15]. Offering virtual attendance options or additional dates for future engagement events in alternative locations may improve participation rates.

Moreover, only 39% of event attendees completed feedback. This limits the generalizability of these results, as this small and self-selected patient cohort could have provided a more positive response which may not be reflective of the overall attitudes of all trial participants or attendees. Though the event organization was well rated, low event feedback rates may be reflective of a lack of time available for attendees to complete this process. In contrast, dedicated time was provided for the completion of the trial experience questionnaires with a high completion rate noted (95.2% [20/21] of trial participants who attended the event). Further events should therefore consider incorporating protected time for event-specific feedback forms to be completed. Furthermore, a specific assessment of the perceived intensity of the event, as a dedicated question within the feedback form, should be included.

Despite these limitations, this end-of-trial engagement event appears to provide a valuable platform for trial participants to reconnect with the study team and discuss shared experiences, and for study team members to provide an update about the study progress whilst promoting education around clinical trials, and to generate new ideas for improved trial design in the future. This paper provides a model on which to build future events which could be adopted in the protocols for future long-term trials, particularly those involving high participant burden such as surgical or cell therapy studies. Future trials should prospectively include similar events within their study protocols and budget. The inclusion of virtual attendance options and incorporating dedicated feedback time may further improve its utility and participant engagement. Our research group has incorporated this design into a future event to engage with the same patients once primary ASCOT trial results are published. This will ensure patients are felt valued, as results will be shared with trial participants first who can help guide how best to disseminate study results.

Although the trial team and authors assumed the burden of the trial to be high, most participants considered it as moderate. This moderate burden may have contributed to the participants reporting the overall trial experience as being highly positive. This is reflected in a high overall experience score and all respondents indicating a willingness to re-participate in the trial. This aligns to current evidence suggesting participants involved in clinical research, not limited to cell therapy trials, generally report a positive research experience [3,16]. The positive experience of the research team reported by trial participants (Supplementary File, S6) may contribute to the high trial experience score, corroborating existing evidence that participant-researcher relationships are key drivers of trial satisfaction and retention [17]. This may in turn be driven by the multidisciplinary nature of the research team [18]. Obi et al. also report high satisfaction rates when evaluating the experience of patients enrolled in early-phase cell therapy trials to manage perianal fistulising Crohn’s disease, with communication between participants and members of the trial team cited as being important in increasing engagement retention [19]. Time commitment and cost were highlighted as key challenges to trial participation in their study [19]. In addition, the positive trial experience reported by participants may be influenced by the sense of shared experience identified from the engagement event. Discussing shared experiences has been shown to be a source of social support in other healthcare settings [20]. The engagement event itself may therefore have influenced participants’ perspectives relating to their trial experience. Future work exploring patient experiences should therefore involve data collection immediately after study involvement to reduce the impact of these external influences.

The reasons for wanting to re-participate, joining and staying in the trial appear similar, centered around wanting to help others and accessing new treatment. These motivations align with recent evidence investigating which factors influence an individual’s decision to participate in randomized clinical trials [21]. Altruism and access to new or existing treatments were cited as key factors with a high confidence. However, there is a complex interaction of associated factors falling within the themes of trial influences (including the trial burden), personal influences (including the influence of friends/family), and impact of potential outcomes (including the potential to benefit others) [21].

In contrast, though only reported by three participants, the most common reason for not wanting to re-participate in the trial was lack of access to individual test results. This was reinforced by responses identifying the return of both personal and overall trial results as important factors when considering future trial participation. These findings suggest an expectation among trial participants for transparency and may reflect the evolving landscape of trial participation, with individuals expecting a more collaborative and informed role rather than remaining passive subjects. A balance is required between maintaining blinding and sharing of individual results, which may enhance trust and engagement in future research. Despite growing recognition of the importance of sharing overall trial results with participants, along with the challenges in doing so [22,23], examples of patient-specific trial results being shared are less well documented. These findings should be applied to all future clinical trials, where potential participants should be clearly informed of what information they will be blinded to, and when to expect unblinding as part of the informed consent process. This information should be documented in the patient information pack and be provided prior to study recruitment to set clear expectations. We suggest regulatory guidelines for research specifically involving Advanced Therapy Medicinal Products (ATMPs) should mandate the development of participant-centered communication plans that explicitly addresses blinding and data-sharing protocols as part of the informed consent process. This will help maintain confidence and societal support for ongoing ATMP development.

The assessment of the rehabilitation experience of trial participants is a novel aspect of this work. Rehabilitation experience for an orthopedic cell therapy is a particularly important aspect to consider because it is part of the overall trial experience and has the potential to directly affect the trial result. Variability in rehabilitation experience was noted, particularly with respect to patient education relating to prehabilitation exercises and time to outpatient physiotherapy appointment. This highlights the difficulties with delivering a highly specialized therapy at a tertiary hospital center, where staff have the relevant experience and expertise, and then relying on participants performing their rehabilitation at centers without this specialized expertise. Previous trials have also highlighted that physically attending outpatient rehabilitation services can be burdensome to patients and their facilities [24,25]. Improving communication and ensuring a well-defined pathway between physiotherapists in secondary and primary care may avoid unnecessary delays in accessing therapy and enhance satisfaction in future trials and for delivery of specialist cell therapies *per se*. To help with this process, future clinical trials involving rehabilitation should have standardized documented regimes that can be easily disseminated and available to all sites where physiotherapy will be delivered. Additionally, detailed methodologies and protocols for rehabilitation of these cell therapy patients need to be developed and disseminated internationally to ensure standardized care of these patients.

Trial participants demonstrated broad agreement for being involved in future scientific research relating to cartilage repair and early osteoarthritis. However, involvement in attending scientific presentations and participating in patient involvement group meetings were associated with high rates of disagreement. Furthermore, although respondents were in agreement that the involvement of patients and the public in all stages of research was important, only a minority reported they would like to themselves be involved as patient representatives in developing future research. This suggests that although patients may be prepared to take an active role in future research as a trial participant engaging in all types of procedures or interventions, the majority are reluctant to engage in processes involving trial development as a patient representative despite recognizing the importance of PPIE. These findings are consistent with current evidence, where multiple barriers affecting the experience of patient and public members as collaborators in research are reported [10] whereas attitudes to PPIE from a health researcher perspective are becoming increasingly positive [26] albeit affected by a complex interplay of workplace factors [27]. These barriers can include insufficient training, lack of information and poorly defined roles or expectations [10]. Furthermore, logistical constraints such as travel to meetings and time to pursue these activities have been identified as potential challenges [28]. A recent systematic review has highlighted recommendations to improve the experience of these individuals by ensuring their contribution in the research process is recognized and demonstrated through regular support, feedback and communication [29].

PPIE in research has been shown to improve the quality of its output [30]. Of note, there is an increasing recognition of involving patients in defining trial outcomes to ensure these accurately measure quality of life, particularly in the context of pragmatic trials [31]. Our study has demonstrated that post-trial questionnaires can help to define participant attitudes to inform future trial delivery. In future questionnaires, patients’ attitudes on trial measures and outcomes could be included for further utility. For example, questions relating to whether participants felt the PROMs or other outcome measures recorded truly reflected their experiences, or whether other clinical or functional metrics may better measure changes in quality of life, could be included. These responses will help the delivery of future trials and should be collected immediately after having been involved in the study to reduce the influence of recall bias.

Although a novel area of research, several limitations exist. Firstly, although the first section of the Trial Participant Clinical Experience Questionnaire was based on the validated RPPS, the second section and other questionnaires were study-specific and have not been externally validated, limiting the reliability and generalizability of these findings. Secondly the response rate (as a proportion of total trial participants) is low and the respondents may be biased toward more engaged or satisfied trial participants. Indeed, of the twenty-six trial participants who completed the questionnaires, twenty attended the engagement event. This overlap suggests responses may be weighted toward trial participants who were already motivated, satisfied and willing to reengage. Thirdly, although no clear differences were identified in the available baseline demographics of trial participants who engaged with the event or questionnaires and those who did not, ethnicity was not recorded in the ASCOT clinical trial dataset. Consequently, comparative analysis was not possible. While questionnaire responses suggest a lack of ethnic diversity, it remains unknown whether this reflects the actual demographic distribution of the full ASCOT trial population or indicates a selection bias in our study. Fourthly, participants were asked to reflect on experiences which may have taken place several years ago. This is particularly true of individuals recruited early in the trial. Results are therefore subject to recall bias and may also be influenced by recent experiences including the engagement event. We have therefore proposed that future work exploring patient experiences should involve data gathered immediately following study involvement. Finally, we acknowledge that the small sample size (twenty-six participants completing the questionnaire) may limit the validity of our findings. However, the primary aim of the analysis was exploratory and descriptive, with the results providing valuable qualitative insights for the design and delivery of future clinical trials relating to cell-based therapies.

## 5. Conclusions

This study provides valuable insights into the experience and perspective of participants involved in a long-term orthopedic cell therapy trial. Despite the substantial demands involved in trial participation, the overall trial experience was highly positive. Key areas identified for improvement in future studies will include early communication with patients regarding sharing of trial results with an understanding of exactly what data will be shared to promote transparency, enhanced communication and coordination of rehabilitation between primary and secondary care, and improving the experience of patients and public members as research collaborators in the PPIE process. The feasibility and positive impact of an end-of-trial engagement event has also been highlighted. This trial participant engagement event provides a framework to follow in future long-term trial designs. However, improved strategies to promote attendance and broader engagement are required.

## Supplementary Material

Supplementary File S2.docx

Supplementary File S4.docx

Supplementary File S3.docx

Supplementary File S6.docx

Supplementary File S1.docx

Supplementary File S5.xlsx

## Data Availability

The data that supports the fundings of this study are not openly available due to reasons of sensitivity. An anonymized dataset may be available from the corresponding author upon reasonable request.

## Article highlights

- Undertaking an end-of-trial engagement event to reengage with cell-based therapy clinical trial participants is feasible and valuable. A framework for this has been presented that could be adopted in future long-term trials. However, strategies to broaden accessibility and promote participant attendance at these events is required.
- Questionnaire-based methods were used to assess the clinical experience of participants who took part in a long-term, cell-based therapy clinical trial. Experiences were highly positive despite the assumed high-burden nature of the study, and all respondents stated they would participate again if given the opportunity.
- Participants value transparency and the sharing of individualized test and trial results. This should be considered in the design of future cell-based therapy clinical trials, with clear communication to set expectations of what data will be shared with participants.
- Improved coordination of rehabilitation services between primary and secondary care is required to improve the variability in rehabilitation experiences. In addition, strategies to encourage patients and public members to engage more actively as representatives in trial development are needed.
- These findings highlight the value of structured PPIE events to improve trial design, participant experience, and future engagement in long-term cell therapy research.
