# Supplementary material for: Evaluating the utility of a patient and public involvement and engagement (PPIE) end-of-trial event to re-engage with cell-based therapy participants
Source: Regen Med. 2025 Dec 18;20(12):673–87. doi: 10.1080/17460751.2025.2601546 (PMC12915861; doi:10.1080/17460751.2025.2601546)
Supplement: Supplementary File S2.docx [file IRME_A_2601546_SM9422.docx]

ASCOT

ASCOT Clinical Trial

Trial Participant Clinical Experience Questionnaire

**Informed Consent Information**

1. The survey that follows aims to understand the experiences of research participants involved in the ASCOT trial.
2. Participation involves completing the survey.

The survey asks about you and your experience of being involved in the ASCOT trial.

It will take 10-15 minutes to complete the survey. Plan to complete the survey in one sitting.

The survey will be offered again following invitation to a research event in December 2023. Re-taking the survey is optional.

1. There are no direct benefits to you. However, the survey results may help to improve the research participation experience in future studies or trials.
2. Information collected in this survey will be held and maintained by The Robert Jones and Agnes Hunt Hospital. Information collected will be used to support other research in the future and may be shared anonymously with other researchers.

We will NOT reveal any individual survey responses in our publications.

We do NOT ask for any details about your prior research studies, doctors, hospitals, or any diagnoses.

1. Your participation is voluntary. There is no compensation for completing the survey.

**Qualification Question**

Have you participated in the ASCOT Clinical Trial? (It does not matter whether you completed, withdrew, or are still enrolled in the study).

No

Yes

**Consent to Participate**

Proceed to the survey?

Yes, I would like to participate in the research by completing the survey.

No, I decline the survey.

**Please answer the questions below regarding the ASCOT trial you participated in.**

**When the survey questions refer to "the study," we are asking about your experiences enrolled in the ASCOT trial, not this survey.**

Would you recommend joining a research study to your family and friends?

Definitely no

Probably no

Probably yes

Definitively yes

Below is a list of possible reasons for joining a research study. When you considered joining the study, how important were these reasons for you?

|  | Very important | Somewhat important | Not very important | Not important at all |
| --- | --- | --- | --- | --- |
| To find out more about my disease |  |  |  |  |
| Because no other medical options were available |  |  |  |  |
| To gain access to new treatment/therapy |  |  |  |  |
| To help others |  |  |  |  |
| Because of the Research/Health Center’s reputation |  |  |  |  |
| Because I am concerned about the topic of study |  |  |  |  |
| To obtain education and learning |  |  |  |  |
| Because of a positive experience in another study |  |  |  |  |
|  | **Very important** | **Somewhat important** | **Not very important** | **Not important at all** |
| Because of family influence/involvement |  |  |  |  |
| Because my caregiver encouraged me |  |  |  |  |
| Any other reasons (please specify) |  | | | |

Did the informed consent process prepare you for what to expect during the study?

No

Yes, somewhat

Yes, mostly

Yes, completely

Did the information and discussions you had before participating in the research study prepare you for your experience in the study?

No

Yes, somewhat

Yes, mostly

Yes, completely

Did the research team members listen carefully to you?

Never

Sometimes

Usually

Always

Did the research team members treat you with courtesy and respect?

Never

Sometimes

Usually

Always

During your discussion about the study, did you feel pressure from the research staff to join the study?

Never

Sometimes

Usually

Always

When you were not at the research site did you know how to reach the research team if you had a question?

Never

Sometimes

Usually

Always

When you were not at the research site and you needed to reach a member of the research team, were you able to reach him/her as soon as you wanted?

Never

Sometimes

Usually

Always

Did not need to reach the research team

Did you feel you were a valued partner in the research process?

Never

Sometimes

Usually

Always

If you considered leaving the study, did you feel pressure from the Research Team to stay?

Never

Sometimes

Usually

Always

Did not consider leaving the study

Below is a list of possible reasons for leaving a research study. How important were the reasons for you in considering leaving the study?

|  | Very important | Somewhat important | Not very important | Not important at all |
| --- | --- | --- | --- | --- |
| Pain or discomfort related to participation |  |  |  |  |
| Worried about risks of treatment |  |  |  |  |
| Side effects that occurred during the study |  |  |  |  |
| Invasion of privacy |  |  |  |  |
| Too much time spent waiting around |  |  |  |  |
| Time commitment required |  |  |  |  |
| Family/work issues unrelated to the study |  |  |  |  |
| Interactions with research team |  |  |  |  |
| Not getting test results |  |  |  |  |
| Undue pressure to stay in study |  |  |  |  |
| Unexpected tests and procedures that occurred during the study |  |  |  |  |
| Transportation/parking |  |  |  |  |
| Any other reasons (please specify) |  | | | |

Please use the scale below to rate you overall experience in the research study, where 0 is the worst possible experience, and 10 is the best possible experience.

0 (Worst)

1

2

3

4

5

6

7

8

9

10 (Best)

Below is a list of possible reasons for staying in a research study. How important were these reasons for you in staying in the research study?

|  | Very important | Somewhat important | Not very important | Not important at all |
| --- | --- | --- | --- | --- |
| To find out more about my disease |  |  |  |  |
| Because no other medical options were available |  |  |  |  |
| To gain access to new treatment/therapy |  |  |  |  |
| To help others |  |  |  |  |
| Because of the Research/Health Center's reputation |  |  |  |  |
| Because I am concerned about the topic of study |  |  |  |  |
| To obtain education and learning |  |  |  |  |
|  | **Very important** | **Somewhat important** | **Not very important** | **Not important at all** |
| Because of a positive experience in another study |  |  |  |  |
| Because of family influence/involvement |  |  |  |  |
| Because my caregiver encouraged me |  |  |  |  |
| Because of my relationship with the research team |  |  |  |  |
| Feeling valued as a research participant |  |  |  |  |
| Improved health or quality of life |  |  |  |  |
| Any other reasons (please specify) |  | | | |

How much did the study demand of you? (Pick the answer that most closely describes your experience).

1. Simple (for example: a few visits or simple tests or surveys)

2. Moderate (for example: multiple visits or a short inpatient stay; only a few procedures, not risky or intense)

3. Intense (for example: long or multiple inpatient stays or many visits; procedure(s) that are intense, risky, or complex)

Which of the following things would be important for you in a future study?

Access to comfortable bed during visits

Travel reimbursement

Availability of support groups for the condition being studied

Flexible schedule

Accessible parking and study location

Planned discharge and proper goodbye to research team

Summary of overall research results shared with me

Results of personal lab tests shared with me

Other (please specify)

______________________________________________________________________

Would you participate in the ASCOT trial again if given the opportunity?

Definitely no

Probably no

Probably yes

Definitively yes

If yes, please select the top three reasons for wanting to participate in the trial again:

To find out more about my disease

Because no other medical options were available

To gain access to new treatment/therapy

To help others

Because of the Research/Health Center’s reputation

Because I am concerned about the topic of study

To obtain education and learning

Because of a positive experience in another study

Because of family influence/involvement

Because my caregiver encouraged me

Other:

If no, please select the top three reasons for not wanting to participate in the trial again:

Pain or discomfort related to participation

Worries about risks of treatment

Side effects that occurred during the study

Invasion of privacy

Too much time spent waiting around

Time commitment required

Family/work issues unrelated to the study

Interactions with research team

Not getting test results

Undue pressure to stay in study

Unexpected tests and procedures that occurred during the study

Transportation/parking

Other:

_________________________________________________________________________

What do you think were the best aspects of the ASCOT trial?

_________________________________________________________________________

_________________________________________________________________________

_________________________________________________________________________

What do you think were the worst or most difficult aspects of the ASCOT trial?

_________________________________________________________________________

_________________________________________________________________________

_________________________________________________________________________

What would you do differently if you were designing a trial like this?

_________________________________________________________________________

_________________________________________________________________________

_________________________________________________________________________

**The following questions seek your opinion on public and patient involvement in future research relating to cartilage repair and osteoarthritis.**

Please read the statements below and tick the box that most accurately describes what you feel.

1. Involving representatives of patients and the general public in the processes of designing a study or clinical trial is important.

Strongly Agree

Agree

Mildly Agree

Mildly Disagree

Disagree

Strongly Disagree

1. Involving representatives of patients and the general public in discussions of how best to publicise trial results is important?

Strongly Agree

Agree

Mildly Agree

Mildly Disagree

Disagree

Strongly Disagree

The stages involved in developing a study or clinical trial have been listed below. How important is it to involve patients and the general public in each of these stages?

|  | Very Important | Somewhat important | Not very important | Not important at all |
| --- | --- | --- | --- | --- |
| Developing research ideas and priorities |  |  |  |  |
| Formulating a specific research question |  |  |  |  |
| Design of a specific study or trial |  |  |  |  |
| Disseminating study findings and results |  |  |  |  |

Would you like to be involved as a patient/public representative in developing future research?

Yes

No

Please could you state why you would or would not like to be involved as a patient/public representative in developing future research?

______________________________________________________________________

______________________________________________________________________

______________________________________________________________________

At each stage, how likely is it you will become involved as a patient/public representative in developing future research?

|  | Very Likely | Likely | Neutral | Unlikely | Very Unlikely |
| --- | --- | --- | --- | --- | --- |
| Developing research ideas and priorities |  |  |  |  |  |
| Formulating a specific research question |  |  |  |  |  |
| Design of a specific study or trial |  |  |  |  |  |
| Disseminating study findings and results |  |  |  |  |  |

**Please answer the questions below relating to your demographic profile.**

What is your age?

18-34

35-44

45-54

55-65

65 and over

What is your gender?

Male

Female

Prefer not to say

Other (please give details)

______________________________________________________________________

Is your gender identity the same as the gender you were assigned at birth?

Yes

No

Prefer not to say

What is your ethnicity?

Asian or Asian British

Black or Black British

Mixed

White

Other ethnic group

Prefer not to say

Please write the postcode of your registered GP Practice:

______________________________________________________________________

Is there anything else you would like to share about your experience in the ASCOT trial?

______________________________________________________________________

______________________________________________________________________

______________________________________________________________________

**THANK YOU FOR COMPLETING THIS QUESTIONNAIRE**
