# Supplementary material for: Evaluating the utility of a patient and public involvement and engagement (PPIE) end-of-trial event to re-engage with cell-based therapy participants
Source: Regen Med. 2025 Dec 18;20(12):673–87. doi: 10.1080/17460751.2025.2601546 (PMC12915861; doi:10.1080/17460751.2025.2601546)
Supplement: Supplementary File S4.docx [file IRME_A_2601546_SM9421.docx]

ASCOT

ASCOT Clinical Trial

Trial Participant Rehabilitation Experience Questionnaire

**Informed Consent Information**

1. The survey that follows aims to understand the rehabilitation experiences of research participants involved in the ASCOT trial.
2. Participation involves completing the survey.

The survey asks about you and your rehabilitation experience as part the ASCOT trial.

It will take 5-10 minutes to complete the survey. Plan to complete the survey in one sitting.

The survey will be offered again following invitation to a research event in December 2023. Re-taking the survey is optional.

1. There are no direct benefits to you. However, the survey results may help to improve the research participation experience in future studies or trials.
2. Information collected in this survey will be held and maintained by The Robert Jones and Agnes Hunt Hospital. Information collected will be used to support other research in the future and may be shared anonymously with other researchers.

We will NOT reveal any individual survey responses in our publications.

We do NOT ask for any details about your prior research studies, doctors, hospitals, or any diagnoses.

1. Your participation is voluntary. There is no compensation for completing the survey.

**Qualification Question**

Have you participated in the ASCOT Clinical Trial? (It does not matter whether you completed, withdrew, or are still enrolled in the study).

No

Yes

**Consent to Participate**

Proceed to the survey?

Yes, I would like to participate in the research by completing the survey.

No, I decline the survey.

**Please answer the questions below regarding the ASCOT trial you participated in.**

**Before your surgery**

Pre-habilitation: Were you given any exercises to improve your fitness and muscle strength before surgery?

Yes

No

Do’s and Don’ts: Were you given specific advice about what activities to avoid doing, or what types of activity would be beneficial following your surgery?

Yes

No

Weight-bearing: Was it explained to you how much weight you would be expected to put through your affected limb after your surgery?

Yes

No

Range of Movement: Were you informed whether the amount of bending and straightening at your knee would be limited following your surgery?

Yes

No

Exercises: Were you given examples of what exercises you would be expected to do after your surgery and how often?

Yes

No

Duration of Rehabilitation: Were you given an indication of how long and how often you would need to comply with physio/rehabilitation?

Yes

No

**After your surgery**

In-Patient Physio: Did you see a physio on the ward after your surgery?

Yes

No

Weight Bearing: Were you taught how to use crutches?

Yes

No

N/A

Range of Movement: Were you advised to limit the amount of bending and straightening at the knee?

Yes

No

N/A

Out-Patient Physio: How long did you have to wait to see a local out-patient physio?

0-2 weeks

3-6 weeks

Over 6 weeks

Discharge: How long were you under a care of a physio before you were discharged?

0-3 months

4-6 months

7-12 months

Over 12 months

**How can we improve?**

Do you think access to an online rehab service would have been helpful?

Yes

No

Can you offer any recommendations to improve how we deliver pre- and post-operative rehabilitation?

______________________________________________________________________

______________________________________________________________________

______________________________________________________________________

**THANK YOU FOR COMPLETING THIS QUESTIONNAIRE**
