# Supplementary material for: Evaluating the utility of a patient and public involvement and engagement (PPIE) end-of-trial event to re-engage with cell-based therapy participants
Source: Regen Med. 2025 Dec 18;20(12):673–87. doi: 10.1080/17460751.2025.2601546 (PMC12915861; doi:10.1080/17460751.2025.2601546)
Supplement: Supplementary File S3.docx [file IRME_A_2601546_SM9420.docx]

ASCOT

ASCOT Clinical Trial

Trial Participant Perspectives for Translational Scientific Research

**Informed Consent Information**

1. The survey that follows aims to understand how you feel about scientific research, experimental treatments and clinical trials for cartilage repair/early osteoarthritis.
2. Participation involves completing the survey.

It will take 5-10 minutes to complete the survey. Plan to complete the survey in one sitting.

The survey will be offered again following invitation to a research event in December 2023. Re-taking the survey is optional.

1. There are no direct benefits to you. However, the survey results may help to improve the research participation experience in future studies or trials.
2. Information collected in this survey will be held and maintained by The Robert Jones and Agnes Hunt Hospital. Information collected will be used to support other research in the future and may be shared anonymously with other researchers.

We will NOT reveal any individual survey responses in our publications.

We do NOT ask for any details about your prior research studies, doctors, hospitals, or any diagnoses.

1. Your participation is voluntary. There is no compensation for completing the survey.

**Qualification Question**

Have you participated in the ASCOT Clinical Trial? (It does not matter whether you completed, withdrew, or are still enrolled in the study).

No

Yes

**Consent to Participate**

Proceed to the survey?

Yes, I would like to participate in the research by completing the survey.

No, I decline the survey.

**Name**: ___________________________________________________________________

**Read the following statements and tick the box that most accurately describes how you feel.**

Statement 1: I am willing to be involved in further scientific research into cartilage repair/early osteoarthritis, which includes:

|  | **Strongly agree** | **Agree** | **Mildly agree** | **Mildly disagree** | **Disagree** | **Strongly disagree** |
| --- | --- | --- | --- | --- | --- | --- |
| Receiving postal communications on scientific research into Cartilage repair/ treatments for early Osteoarthritis |  |  |  |  |  |  |
| Attending scientific presentations |  |  |  |  |  |  |
| Participating in more questionnaires |  |  |  |  |  |  |
| Participating in patient involvement group meetings |  |  |  |  |  |  |
| Consenting for scientific researchers to access my patient records |  |  |  |  |  |  |
| Donating blood for research purposes |  |  |  |  |  |  |
| Donating saliva for research purposes |  |  |  |  |  |  |
| Donating fat for research purposes |  |  |  |  |  |  |
| Donating bone marrow for research purposes |  |  |  |  |  |  |
| Donating fat pad (fat from within the knee joint) for research purposes |  |  |  |  |  |  |
| Donating synovium (a membrane which surrounds the knee joint) for research purposes (whilst under general anaesthetic in surgery) |  |  |  |  |  |  |

|  | **Strongly agree** | **Agree** | **Mildly agree** | **Mildly disagree** | **Disagree** | **Strongly disagree** |
| --- | --- | --- | --- | --- | --- | --- |
| Donating synovium (a membrane which surrounds the knee joint) for research purposes (during an out-patient appointment under ultrasound, with local anaesthetic). N.B. a small skin incision (3-4mm; roughly the width of a matchstick) would need to be made on your knee |  |  |  |  |  |  |
| Donating synovial fluid samples for research purposes (whilst under general anaesthetic in surgery) |  |  |  |  |  |  |
| Donating synovial fluid samples for research purposes (during an out-patient appointment under ultrasound, with local anaesthetic) |  |  |  |  |  |  |
| Donating surgical waste tissues |  |  |  |  |  |  |

Any other comments?

_________________________________________________________________________

_________________________________________________________________________

_________________________________________________________________________

Statement 2: I would consider taking part in an experimental treatment or clinical trial that involved the use of:

|  | **Strongly agree** | **Agree** | **Mildly agree** | **Mildly disagree** | **Disagree** | **Strongly disagree** |
| --- | --- | --- | --- | --- | --- | --- |
| Cells derived from animal tissues |  |  |  |  |  |  |
| Cells derived from  human embryos *(up to 8 weeks old)* |  |  |  |  |  |  |
| Cells derived from human foetuses *(up to 4 months old)* |  |  |  |  |  |  |
| Cells derived from human umbilical cords/ placentas |  |  |  |  |  |  |
|  | **Strongly agree** | **Agree** | **Mildly agree** | **Mildly disagree** | **Disagree** | **Strongly disagree** |
| Cells derived from deceased adult human donor tissues *(a stranger)* |  |  |  |  |  |  |
| Cells derived from adult human donor tissues *(a friend or relative)* |  |  |  |  |  |  |
| Cells derived from living adult human donor tissues *(a stranger)* |  |  |  |  |  |  |
| Cells derived from my own tissues |  |  |  |  |  |  |
| Cells that have been genetically modified |  |  |  |  |  |  |
| New experimental drugs |  |  |  |  |  |  |

Any other comments?

_________________________________________________________________________

_________________________________________________________________________

_________________________________________________________________________

Statement 3: I would probably be prepared to take part in an experimental treatment or clinical trial which had NOT first been tested on:

|  | **Strongly agree** | **Agree** | **Mildly agree** | **Mildly disagree** | **Disagree** | **Strongly disagree** |
| --- | --- | --- | --- | --- | --- | --- |
| Other people |  |  |  |  |  |  |
| Large laboratory animals (more similar to humans) e.g. sheep/ horse |  |  |  |  |  |  |
| Laboratory rodents |  |  |  |  |  |  |
| Any laboratory animals |  |  |  |  |  |  |

Any other comments?

_________________________________________________________________________

_________________________________________________________________________

_________________________________________________________________________

Statement 4: In order to monitor whether an experimental treatment or clinical trial (before, during and after) was making a difference I would probably be prepared to take part in:

|  | **Never** | **Once (after 12 months)** | **Monthly (for 12 months)** | **Weekly (for 12 months)** | **Whatever the trial might require** |
| --- | --- | --- | --- | --- | --- |
| Physical therapy |  |  |  |  |  |
| MRI imaging |  |  |  |  |  |
| CT imaging |  |  |  |  |  |
| X-ray imaging |  |  |  |  |  |
| PET scans  These require injection of a radiotracer injected into a vein. |  |  |  |  |  |
| Completing patient reported outcome scores e.g. Lysholm, PANAS, HAP, ICOAP |  |  |  |  |  |
| Donating a biopsy of cartilage from the site used to harvest tissue for Autologous Chondrocyte Implantation (ACI). N.B. This would require an additional arthroscopic surgery 12 months post-treatment. |  |  |  |  |  |
| Donating a biopsy of cartilage from the Autologous Chondrocyte Implantation (ACI) repaired tissue site. N.B. This would require an additional intervention 12 months post-treatment. |  |  |  |  |  |
| Keeping a diary/ log book |  |  |  |  |  |
| Activity monitoring (smart watch/ electronic data) |  |  |  |  |  |
| Using a mobile phone app (e.g. MyRecovery) to watch videos about how to do your physio and to log your rehab activity |  |  |  |  |  |
| Using a mobile phone app to enter questionnaire scores (the app would send alerts when you need to complete scores) |  |  |  |  |  |

|  | **Never** | **Once (after 12 months)** | **Monthly (for 12 months)** | **Weekly (for 12 months)** | **Whatever the trial might require** |
| --- | --- | --- | --- | --- | --- |
| GAIT analysis (attending appointments in a specialist laboratory to monitor how you walk/move) |  |  |  |  |  |

Any other comments?

_________________________________________________________________________

_________________________________________________________________________

_________________________________________________________________________

Statement 5: I would be happy for my cells/tissues/samples to be used for:

|  | **Strongly agree** | **Agree** | **Mildly agree** | **Mildly disagree** | **Disagree** | **Strongly disagree** |
| --- | --- | --- | --- | --- | --- | --- |
| Genetic testing |  |  |  |  |  |  |
| Animal studies |  |  |  |  |  |  |
| Research studies at other UK centres |  |  |  |  |  |  |
| Research studies at other international centres |  |  |  |  |  |  |

Any other comments?

_________________________________________________________________________

_________________________________________________________________________

_________________________________________________________________________

Statement 6: I would probably take part in an experimental treatment or clinical trial if it was a 50:50 chance whether I received the trial drug/cell or a placebo. *(A placebo is, for example a tablet which looks the same but does not contain any active drug)*

Strongly Agree

Agree

Mildly Agree

Mildly Disagree

Disagree

Strongly Disagree

Statement 7: I would like to be kept updated about future research performed.

Yes – all research from the group

Yes – only ASCOT related research

No

If yes, I would be interested in hearing about it in the following ways:

|  | **Yes** | **No** |
| --- | --- | --- |
| Email – as and when research is available |  |  |
| Email – regular newsletter giving updates on all research in the group (e.g. every 6 months) |  |  |
| Regular newsletter by post giving updates on all research in the group (e.g. every 6 months) |  |  |
| Social media updates (e.g. Twitter, Facebook, Instagram, etc…) |  |  |
| Attending scientific meetings or events – online |  |  |
| Attending scientific meetings or events – in person |  |  |

Any other comments?

_________________________________________________________________________

_________________________________________________________________________

_________________________________________________________________________

**THANK YOU FOR COMPLETING THIS QUESTIONNAIRE**
