# Supplementary material for: Evaluating the utility of a patient and public involvement and engagement (PPIE) end-of-trial event to re-engage with cell-based therapy participants
Source: Regen Med. 2025 Dec 18;20(12):673–87. doi: 10.1080/17460751.2025.2601546 (PMC12915861; doi:10.1080/17460751.2025.2601546)
Supplement: Supplementary File S6.docx [file IRME_A_2601546_SM9419.docx]

Trial Participant Experience of the Research Team

The trial participant experience of the research team appears positive – 96.2% (25/26) reported the research team members either ‘Usually’ or ‘Always’ listened, 92.3% (24/26) were ‘Usually’ or ‘Always’ made to feel a valued partner in the research process, and all respondents reported the research team members ‘Usually’ or ‘Always’ treated the trial participant with courtesy and respect.


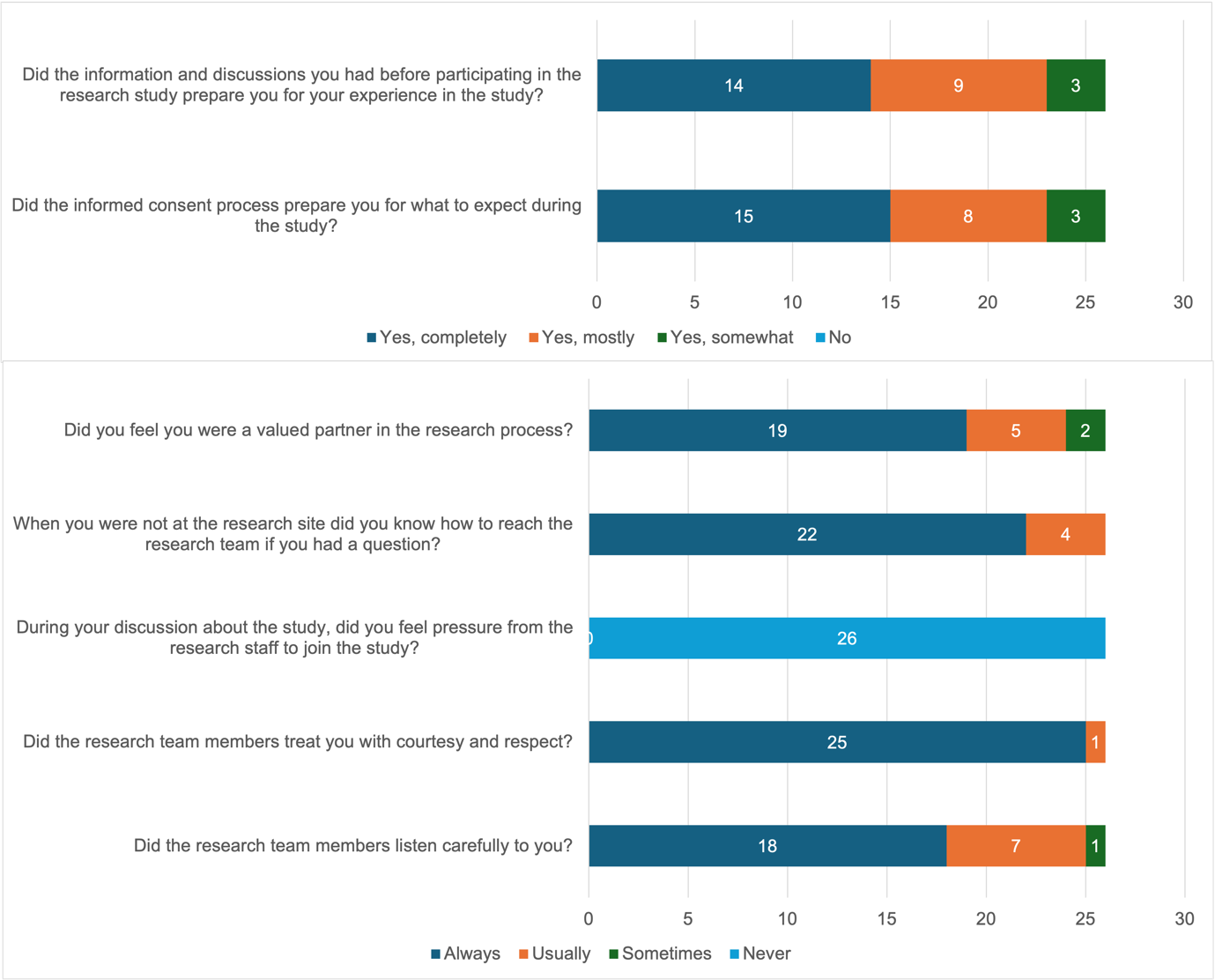


*Responses to questions concerning the informed consent process and interactions with the research team.*
