# Supplementary material for: Evaluating the utility of a patient and public involvement and engagement (PPIE) end-of-trial event to re-engage with cell-based therapy participants
Source: Regen Med. 2025 Dec 18;20(12):673–87. doi: 10.1080/17460751.2025.2601546 (PMC12915861; doi:10.1080/17460751.2025.2601546)
Supplement: Supplementary File S1.docx [file IRME_A_2601546_SM9418.docx]

1. How satisfied were you with ‘Session 1: Trial Development and Delivery’?*
2. Session 1: Comments/Suggestions (Free-Text)
3. How satisfied were you with the tour of our facilities?*
4. Tour: Comments/ Suggestions (Free-Text)
5. How satisfied were you with ‘Session 2: Trial Outcome Measures’?*
6. Session 2: Comments/ suggestions (Free-Text)
7. How satisfied were you with ‘Session 3: Scientific Investigations’?*
8. Session 3: Comments/ suggestions (Free-Text)
9. How satisfied were you with the organisation of the event?*
10. Organisation: Comments/ suggestions (Free-Text)
11. Overall, did you find the celebration to be an enjoyable and engaging experience?*
12. Any other comments/suggestions (Free-Text)

*Rated on a ten-point scale. 1 = not at all; 10 = completely satisfied

*Supplementary File 1 – Questions included in the ASCOT Trial Participant Engagement Event feedback form*
